# SHARP: a hybrid metaheuristic approach for intelligent robotic path planning

**DOI:** 10.1038/s41598-026-54881-7

**Published:** 2026-05-31

**Authors:** Hussam Fakhouri, Sadi Alawadi, Alexander Galozy, Fahed Alkhabbas, Majed Ayyad, Imad Banihani, Khalid Alkharabsheh, Mohammad Aladwan

**Affiliations:** 1 Faculty of Artificial Intelligence, Al-Balqa Applied University, Al-Salt, 19117 Jordan; 2 Department of Computer Science, Blekinge Institute of Technology, Karlskrona, Sweden; 3 School of Information Technology, Halmstad University, Halmstad, Sweden; 4 Department of Computer Science and Media Technology, Malmö University, Malmö, Sweden; 5 Department of Computer Science, Birzeit University, Birzeit, Palestine; 6 Department of Statistics and Business Analytics, United Arab Emirates University, Al Ain, United Arab Emirates; 7 Department of Cyber Security, Jerash University, Jerash, Jordan

**Keywords:** Swarm intelligence, Artificial intelligence, Optimization, Robot path planning, Engineering, Mathematics and computing

## Abstract

Robotic path planning is a fundamental requirement for autonomous navigation, where a robot must reach a target while avoiding obstacles and producing a feasible, smooth, and efficient trajectory. This paper presents SHARP, a hybrid metaheuristic planning framework based on Particle Swarm Optimization, Sine Cosine search, and lightweight Nelder–Mead simplex refinement. The proposed framework introduces two scalarization-based multi-criteria decision layers for robotic path planning: Priority-PSN, which prioritizes path length while penalizing obstacle and boundary violations, and No-Preference-PSN, which selects a balanced solution by minimizing the normalized distance to an ideal point. Cubic-spline interpolation is further applied to convert optimized waypoints into smoother executable trajectories. The approach is validated in static and dynamic two-dimensional environments with different obstacle densities and motion patterns. In six static benchmark environments, PSN consistently produces shorter collision-free paths than PSO, GWO, and SCA. Additional ablation and function-evaluation-normalized experiments show that the full hybrid improves average path quality in cluttered maps, although this improvement is accompanied by higher computational cost. In dynamic replanning experiments, PSN achieves the highest success rate among the evaluated variants, but with increased latency. SHARP provides a practical and adaptable optimization-based framework for intelligent robotic path planning under static and dynamic constraints.

## Introduction

Robotic path planning is a core capability for autonomous navigation, where a robot must move from a start position to a target while avoiding collisions and respecting workspace constraints^1^. In realistic deployments, the problem is inherently *multi-objective*: beyond reaching the goal, planners must typically minimise travel distance, time, or energy, maintain safety with respect to obstacles, and produce trajectories that are smooth enough to be executed by the robot, often under strict computational and real-time requirements^2,3^. These coupled requirements make path planning difficult in cluttered environments, where the search space is highly non-linear and feasibility constraints (collision avoidance) dominate the optimisation landscape.

Swarm intelligence (SI) is a well-established subfield of Artificial Intelligence (AI) that studies collective behaviour emerging from decentralised, self-organised systems. SI has been particularly influential in optimisation, where populations of simple agents cooperate through local interactions to explore complex search spaces^4,5^. Since its introduction in the context of cellular robotics^6^, SI-based optimisation has demonstrated strong robustness and flexibility across a wide range of problems, despite lacking deterministic guarantees of global optimality in difficult landscapes^7^. Among SI methods, Particle Swarm Optimisation (PSO) and its variants are widely used in robotics due to their derivative-free nature and their ability to handle non-convex objective functions.

Multi-objective optimisation (MOO) provides a principled framework for handling competing objectives by seeking trade-off solutions (e.g., Pareto-optimal solutions) rather than a single optimum^8,9^. This perspective aligns naturally with robotic navigation, where improving one criterion, such as path length, can have an adverse effect on another, for example, the safety margin. Consequently, combining MOO with SI metaheuristics is an attractive direction for robotic path planning, offering a flexible search behaviour while accommodating multi-criteria decision-making^3^.

However, many existing planning pipelines either (i) optimise a single composite cost using fixed weighted sums, which makes the outcome sensitive to hand-tuned weights and may bias the solution toward one objective, or (ii) apply SI methods such as standard PSO that can lose diversity and converge prematurely in obstacle-dense environments, leading to longer paths or infeasible solutions when constraints are tight^2,10^. These limitations motivate lightweight multi-objective formulations that remain practical for robotic planning while improving robustness in cluttered spaces.

To address these challenges, this paper focuses on *multi-objective extensions* of the PSN/PSN-based planning framework for intelligent robot navigation. Specifically, we introduce two complementary variants:

- Priority-PSN: a preference-driven formulation that treats path length as the dominant objective while enforcing obstacle avoidance through a penalty-based cost model.
- No-Preference-PSN: a preference-free formulation that selects a compromise solution by minimising the distance to an ideal point in the objective space, thereby avoiding the need for user-specified weights while still capturing trade-offs among objectives.

Additionally, cubic-spline interpolation is used to smooth the waypoint sequence and generate execution-ready trajectories. The proposed multi-objective variants are evaluated in static 2D workspaces with varying obstacle densities and are compared against PSO, Grey Wolf Optimizer (GWO), and the Sine Cosine Algorithm (SCA), with further demonstration in a dynamic simulation involving moving obstacles and a moving target.

Furthermore, this paper provides a novel shared hybrid search strategy with two scalarization-based decision layers, rather than in a Pareto-archive-based multi-objective optimizer

The remainder of this paper is organized as follows. Section Related work presents an overview of the background and related work on robot path planning and swarm-based optimization techniques. Section SHARP: our approach introduces the proposed multi-objective variants, namely Priority-PSN and No-Preference-PSN. Subsequently, Section Validation describes the experimental setup and reports the results and discussion for both static and dynamic environments. Finally, Section Conclusion and future works concludes the paper and outlines directions for future research.

## Related work

Robot path planning has been approached through classical search, learning-based decision making, and bio-inspired optimisation, with recent work emphasising robustness in dynamic, uncertain, and human-shared environments.

Heuristic search remains attractive due to its interpretability and predictable behaviour, and recent research has improved performance through richer environmental representations and cost modelling. Quan et al. address greenhouse navigation in weakly structured and GPS-denied settings by combining semantic segmentation, visual-semantic fusion SLAM, and a semantically constrained A* planner ^11^. Liu and Xu propose a CNN-VSLAM pipeline that couples deep perception with SLAM for navigation and obstacle avoidance ^12^. For rescue scenarios spanning multiple map conditions, Zhang et al. modify A* using expected-cost fusion across maps and local obstacle replacement to preserve feasibility without full replanning ^13^. These approaches provide strong interpretability and constraint handling, but they can be sensitive to mapping quality and may require careful modelling of costs and environment structure to remain effective across heterogeneous conditions.

Reinforcement learning (RL) is frequently used when obstacle motion, sensing noise, or partial observability make purely geometric planning brittle. Hu et al. combine Conflict-Based Search with a transfer-learning-enhanced TD3 policy for multi-robot navigation, coordinating global conflict resolution with learning-based local avoidance ^14^. Lin et al. improve TD3 with prioritised replay and an LSTM module to better capture temporal dependencies ^15^. Nguyen et al. propose depth denoising and sub-goal guidance for distributional RL navigation in complex unknown environments ^16^. Safety-aware and perception-aware learning has also been explored for human-shared and degraded-sensing settings ^17,18^. While RL can yield strong adaptivity, it typically incurs training costs, sim-to-real considerations, and sensitivity to reward design, which motivates the development of complementary optimisation-based planners for scenarios requiring lower deployment complexity.

In parallel, metaheuristic planning approaches have been developed to balance global exploration with feasible local motion. Recent works improve optimisation performance via enhanced bio-inspired algorithms and hybrid global–local frameworks, e.g., SSTDBO ^19^, ICPO ^20^, and cooperative strategies for dynamic settings ^21^. Hybrid pipelines that combine a global optimiser with a local reactive method (e.g., ACO+DWA) are also common in applied robotics ^22,23^. Earlier studies have explored hybrid PSO variants and other evolutionary approaches for planning, showing that hybridisation can improve robustness in obstacle-rich environments^24–27^. However, many metaheuristic planners still optimise a single composite objective, and their effectiveness can depend heavily on penalty tuning and weight selection, particularly when objectives conflict.

The integration of MOO with swarm intelligence has gained attention^28^, yet the adoption of multi-objective SI in robotic path planning remains comparatively limited^29,30^. Existing solutions often either (i) reduce MOO to a weighted-sum objective, which requires careful weights tuning based on problem complexity, or (ii) maintain a set of Pareto-optimal solutions, which can increase computational and decision overhead when a single executable path is needed.

In contrast to prior work that typically relies on a single scalarisation strategy, this paper introduces two complementary multi-objective planning variants on top of the same PSN/PSN-based search mechanism: *Priority-PSN*, which formalises preference by emphasising path length while enforcing obstacle avoidance through penalties, and *No-Preference-PSN*, which selects a balanced compromise by minimising distance to an ideal point in objective space. This dual formulation supports both preference-aware and preference-free deployment scenarios without requiring redesign of the underlying optimiser. In addition, we employ cubic-spline interpolation to improve the smoothness and executability of the final planned trajectory.

## SHARP: our approach

**Figure 1:**
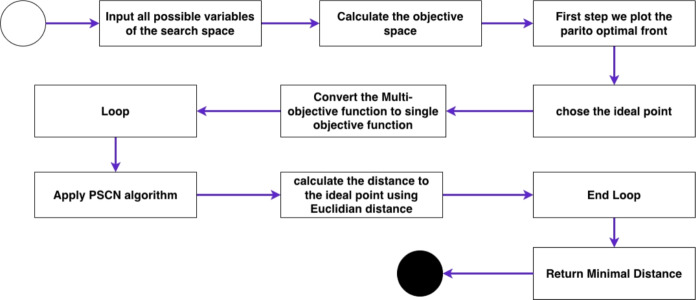
No-preference PSN for solving multi-objective optimization problems.

**Figure 2:**
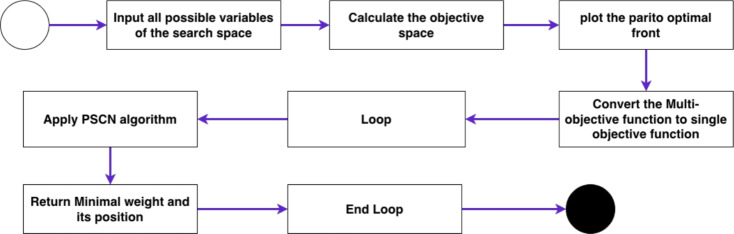
P-PSN for solving multi-objective optimization problems.

This section presents two PSN based algorithms for Multi-Objective Optimization. The first algorithm is named Priority-PSN (see Fig. 2), while the second is named No-Preference-PSN (see Fig. 1). Comprehensive descriptions of both algorithms are provided in the following subsections.

For the specific path-planning problem considered here, both variants operate on the same two normalized criteria: a path-length term $J_1(\textbf{x})=L(\textbf{x})/\Vert \textbf{g}-\textbf{s}\Vert _2$ and a safety term $J_2(\textbf{x})$ that aggregates obstacle and boundary violations. Priority-PSN uses the scalarization $S_{\textrm{P}}(\textbf{x})=J_1(\textbf{x})+\beta J_2(\textbf{x})$, whereas No-Preference-PSN uses the distance-to-ideal scalarization $S_{\textrm{NP}}(\textbf{x})=\Vert [J_1(\textbf{x}),J_2(\textbf{x})]^\top -[1,0]^\top \Vert _2$. Accordingly, we used the term *multi-criteria decision layer* rather than claiming that the algorithm approximates the full Pareto set.

Multi-objective optimization involves multiple, potentially competing objectives, whereas single-objective optimization ranks solutions by a single objective value. Although the concepts and formulations of multi-objective optimization have been delineated in Section 4.1, it is pertinent to reiterate the formulation of the multi-objective optimization problem, presented in Eq. (1).

1 $$\begin{aligned} \min _{x} \Bigl ( f_1(\textbf{x}), f_2(\textbf{x}), \dots , f_m(\textbf{x}) \Bigr ) \end{aligned}$$

where the integer *m* is the number of objectives, and the set *X* is the feasible set of decision vectors.

### No-preference PSN

In the initial phase of the non-preference PSN algorithm applied to multi-objective optimization problems, the multi-objective function is transformed into a single-objective problem. This is achieved by formulating the multi-objective function to yield a singular goal: the distance. The primary objective then becomes minimizing this newly defined single objective. The fitness function computes the distance to the ideal point, which is to be minimized (see Fig. 1). The goal is to identify a solution proximate to the ideal point, as represented in Eq. (2). The overarching goal is to

2 $$\begin{aligned} g(x) = \left\| f(x) - P_\text {ideal} \right\| \end{aligned}$$

where $\vec {x} \in X$, $f(\vec {x}) = [f_1(\vec {x}), f_2(\vec {x}), f_3(\vec {x}), \ldots , f_n(\vec {x})]$, the set $\vec {x}$ includes all the feasible solutions, and $\Vert \cdot \Vert$ is any $L_p$ norm.

The ideal-point rule is instantiated with the normalized pair $(J_1,J_2)$, so the ideal point is $[1,0]^\top$: unit normalized path length and zero violation. This makes the role of the no-preference layer explicit and avoids overstating it as a Pareto-search mechanism. The No-Preference PSN algorithm’s procedure is delineated in Fig. 1. Initially, the algorithm ingests all potential variables within the search space to compute the objective space. Subsequently, the Pareto-optimal front is constructed, encompassing values from the computed objective space. An ideal point, $P_{ideal}$ (e.g., 0,0), is selected from the Pareto-optimal front based on proximity to the ideal point. The fitness function computes the distance to $P_\text {ideal}$ with the intent to minimize this distance. PSN ingests the distance throughout its iterations and converges towards the minimal point in the objective space most proximate to $P_\text {ideal}$. Consequently, this algorithm translates the multi-objective problem into a single-objective problem centred on distance. The final stage returns the minimal distance. As shown in Figure 1, the first step is to introduce the objective space, followed by plotting the Pareto-optimal front that encompasses all selectable values. The subsequent phase entails formulating the fitness function, which selects the objectives to be minimized. The output is the distance to the ideal point, which represents the optimal balance between cost and time. Our methodology emphasizes selecting the optimal minimal value. By deploying the no-preference-PSN algorithm, the system iteratively evaluates each value, applies the fitness function, and determines the fitness value (minimal distance) for selection within the objective space.

In this methodology, we combine the multi-objective targets into a singular objective utilizing the weighted sum approach, as depicted in Eq. (3) :

3 $$\begin{aligned} Minimize: F(x)= w_1 f_1 (x)+ w_2 f_2 (x)+ w_3 f_3 (x)+\cdots w_n f_n (x) \end{aligned}$$

where $w_1+w_2+w_3 +\cdots +w_n = 1$ and *n* is the number of objectives. The fitness function determines the weighted sum of all weighted variables. We are looking for the best solution based on the weight given.

The weighted formulation reduces to a penalty scalarization in which path length is the primary term and collision/boundary violation is penalized through $\beta$. We refers to Priority-PSN as a scalarized multi-criteria planner, not as a Pareto-front method.

### Priority PSN (P-PSN)

The procedural steps of the Priority-PSN algorithm are shown in Fig. 2. The algorithm starts by ingesting all potential variables from the search space to determine the objective space. Subsequently, the Pareto-optimal front is constructed, encompassing the computed objective space values. Within the P-PSN framework, the fitness function computes the aggregate of objective functions by incorporating weights. P-PSN assimilates the weight during each iteration and converges to the minimum objective. Notably, the multi-objective formulation in the P-PSN algorithm is transformed into a single-objective formulation centred on distance. The final phase involves returning the minimum objective.

The algorithm starts by generating a set of random paths within a predefined workspace, called particles. Each particle’s initial velocity is set to zero, and its ’cost’ a measure of the path’s length and its ability to avoid obstacles is calculated. The algorithm’s main loop then iterates over a set number of steps. Within each iteration, the algorithm performs three key updates for every particle in the population. First, PSO updates each particle’s velocity and position based on its own best-known position and the globally best-known position among all particles, thereby serving as a global search mechanism. Second, SCA fine-tunes the particle positions using trigonometric functions, providing a more nuanced navigation strategy in complex spaces. Third, the Nelder-Mead Simplex Method is employed for local optimization, enabling efficient navigation of more intricate terrain. After these updates, the cost associated with each particle is recalculated, and the best-known positions are updated accordingly. The algorithm also adjusts the PSO inertia weight to transition the search strategy from exploration to exploitation as the iterations progress. Upon reaching the maximum number of iterations, the algorithm terminates and returns the path associated with the best-known global position as the optimal solution. This hybrid approach aims to combine the strengths of global and local search algorithms to efficiently solve the robot path planning problem while avoiding obstacles.

To assure fairness of the computational comparison. one PSO update costs one objective-function evaluation per particle, the trigonometric exploration stage adds two probes (sine and cosine candidates), and the simplex-inspired local refinement stage adds three probes (reflection, expansion, and contraction). Hence, the per-iteration evaluation budgets are shown in Eq. 4

4 $$\begin{aligned} \textrm{NFE}_{\textrm{PSO}} = N_{\textrm{pop}}T,\qquad \textrm{NFE}_{\mathrm {PSO+SCA}} = 2N_{\textrm{pop}}T,\qquad \textrm{NFE}_{\textrm{PSN}} = 6N_{\textrm{pop}}T. \end{aligned}$$

## Validation

This section introduces the experiments we conducted to validate the feasibility of SHARP in addressing the complexities of robot path planning. Enhanced by PSN’s capabilities, robots can identify paths more efficiently. The hybrid PSN algorithm aims to minimize the trajectory length (see Algorithm 1). A crucial goal of robot path planning in environments with many obstacles is to ensure a collision-free route, enabling the robot to navigate to the designated target point without colliding with existing obstacles.

**Algorithm 1:**
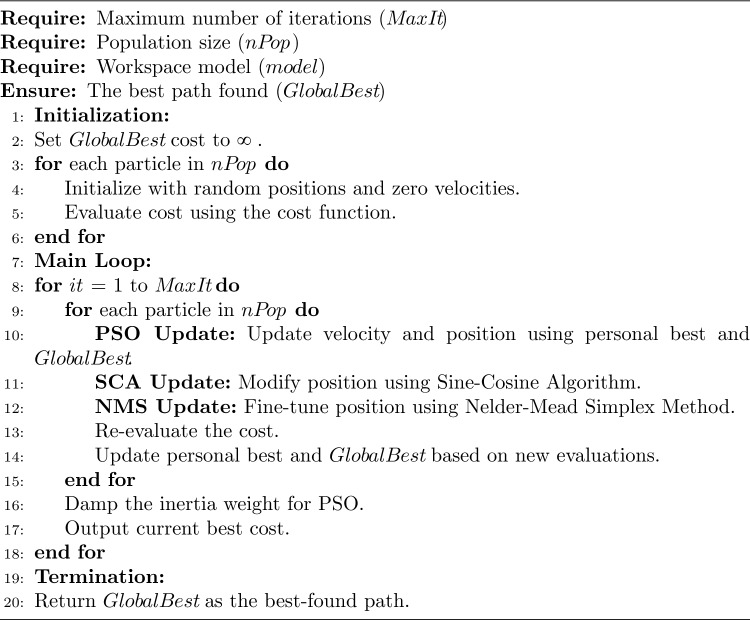
Hybrid PSN for robot path planning.

We conduct **two main experiments** with a sensitivity study to validate SHARP.

**Experiment 1 (planning in static environments)** evaluates collision-free shortest paths in six 2D maps (3–20 fixed obstacles) and benchmarks PSN against PSO, GWO, and SCA.

Sensitivity (Node count) varies the number of intermediate waypoints (N=2–10) to assess robustness to path discretization and to justify the chosen representation before spline smoothing.

**Experiment 2 (planning in dynamic environments)** tests online re-planning with moving obstacles and a moving goal, demonstrating adaptability under time-varying constraints.

Furthermore, we perform an ablation study that compares PSO, PSO+SCA, and the full PSN hybrid. Second, we normalize the comparison by the total number of function evaluations (NFEs), in addition to the fixed-iteration setting. Third, we report runtime, latency, convergence, and success-rate metrics alongside path length. The added static experiments use two representative cluttered maps (Map-S1 and Map-S2), 12 random seeds, population size 30, 100 nominal iterations, five internal path nodes, and $\beta =60$. The added dynamic experiment uses 10 seeds, a population size of 18, 30 replanning iterations per control cycle, five path nodes, and $\beta =80$.

Moreover, We report both fixed-iteration results and equal-NFE results. We also clarify that the local stage is a lightweight simplex-inspired refinement rather than a full inner Nelder–Mead solve to convergence

### Experiment 1: robot path planning in static environment

This experiment evaluates SHARP in a static 2D workspace, where the robot must reach a fixed goal while avoiding stationary circular obstacles. We report the experimental setup, objective and constraints, then compare PSN against PSO, GWO, and SCA in terms of the minimum collision-free path length across multiple obstacle configurations.

#### Experimental setup

Robot path planning in static environments involves determining the robot’s subsequent position based on its current location and a set of predefined environmental constraints. To address this problem, several foundational assumptions are adopted to formalize the planning framework, as listed below:

1. The robot’s initial position and target destination are known a priori.
2. The robot operates within a predefined and bounded search space.
3. The target location is fixed, and the robot continuously navigates toward it.
4. All obstacles in the environment have predetermined and fixed positions prior to the start of each planning phase, consistent with a static environment.
5. Obstacles are modeled as circular objects, allowing for possible overlaps or adjacency between multiple obstacles.
6. The PSN-based path planning process continues iteratively until the robot reaches a predefined proximity threshold relative to the target.

During the experimental implementation, a set of guiding principles is applied in accordance with the above assumptions. Initially, the robot attempts to follow the shortest and most direct path toward the goal. However, when a potential collision with static obstacles is detected along this direct trajectory, the robot invokes the hybrid PSN algorithm to compute an alternative optimal path that avoids collisions. To prevent imminent collisions, the robot dynamically adjusts its navigation route using the PSN algorithm’s multi-objective optimisation, enabling safe traversal through the environment’s feasible regions.

Within the PSN framework, an arbitrary position, denoted as $s(i), m(i), \ldots , X_g(i)$ is assigned as the robot’s initial state within the search domain. The particles in the population represent discrete candidate solutions, and their interactions are governed by distance-based relationships defined over the robot’s positional space.

**Figure 3:**
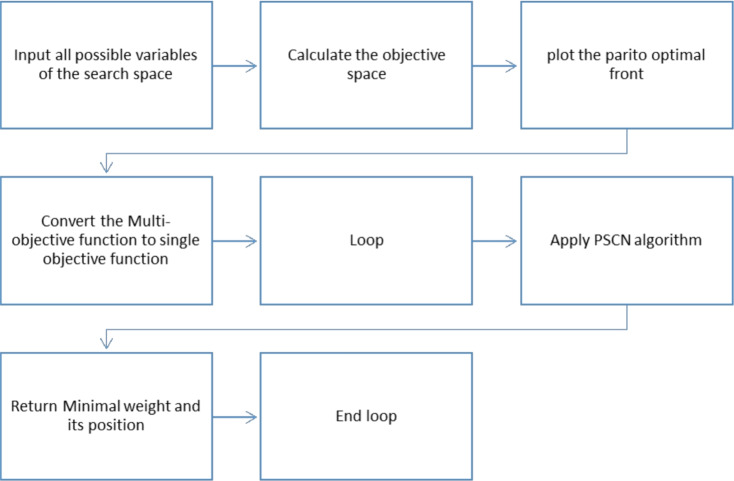
Position of the $X$th particle of the robot path problem.

Let $(X_s(i), Y_s(i))$ represent the robot’s coordinates at iteration $t$, $(X_m(i), Y_m(i))$ denote the intermediate coordinates corresponding to the robot’s position at time $t+1$, and $(X_g(i), Y_g(i))$ indicate the goal location, as illustrated in Figure 3. The determination of the intermediate position $(X_m(i), Y_m(i))$ is achieved through the careful formulation of the objective function, which balances path optimality and obstacle avoidance.

Each particle’s position is defined as a point in a two-dimensional solution space and represents a potential solution to the path planning problem. The PSN algorithm is initialized with a population of particles randomly distributed within the search space and assigned random velocities. These particles are initially concentrated in the vicinity of the robot’s starting position $(X_s(i), Y_s(i))$, facilitating efficient exploration and convergence toward an optimal, collision-free path.

#### Objective function

The optimization problem is formulated around an objective function that minimizes the distance between each robot’s particles and their corresponding target positions. This distance is computed from the robot’s current position to its intended target location, effectively quantifying the length of the robot’s trajectory. Formally, the path that connects particle pairs $(X_s(i), Y_s(i))$, $(X_m(i+1), Y_m(i+1))$, and $(X_g(i), Y_g(i))$ is calculated to ensure that the total length is minimized, subject to the constraint of avoiding any obstacles.

The robot chooses the coordinates $(X_m(i+1), Y_m(i+1))$ to avoid potential collisions with obstacles and to determine its subsequent position. These coordinates are obtained by solving a set of multi-objective PSN equations.

The objective function aims to optimize the path length while ensuring it avoids obstacles. These dual objectives render the problem a candidate for multi-objective optimization. However, the presented algorithmic framework converts this multi-objective problem into a single-objective optimization problem by employing a composite objective function. The transformation is achieved via a penalty-based approach, a common optimization methodology for handling constraint violations.

Specifically, the composite objective function, denoted as $\text {Cost}$, is formulated as follows:

$$\text {Cost} = L \times \left( 1 + \beta \times \text {Violation} \right)$$

where $L$ is the Euclidean length of the proposed path, $\text {Violation}$ quantifies the extent to which the path infringes upon defined obstacles, and $\beta$ is a penalty factor that arbitrates the trade-off between the two objectives.

The variable $L$ is computed by integrating the Euclidean distance over the continuous curve representing the path. It serves as a measure of the ’cost’ of the path in terms of distance travelled. On the other hand, $\text {Violation}$ is a measure calculated based on the average infringement upon obstacles along the path, normalized by the radius of each obstacle. This creates a continuous measure of constraint violation, facilitating the use of gradient-based or gradient-free optimization algorithms.

The penalty factor $\beta$ serves as a scaling coefficient that modulates the relative importance of obstacle avoidance to the path length. A higher value of $\beta$ increases the cost of obstacle violations, thereby compelling the optimization algorithm to prioritize more conservative paths that place greater emphasis on obstacle avoidance. Conversely, a lower value of $\beta$ would make the algorithm more lenient toward slight obstacle infringements in favour of shorter paths.

Through the combination of $L$ and $\text {Violation}$ into a single $\text {Cost}$ function, the algorithm performs what is essentially a weighted sum optimization. This method enables single-objective optimization algorithms, such as Particle Swarm Optimization, while accounting for the multiple facets of the robotic path planning problem.

#### Constraints

Obstacles within the environment are assigned particular values to signify their importance. These obstacles can vary in geometric shape and may overlap. A fundamental assumption underlying this model is that these obstacles are stationary entities. To incorporate them effectively into the path planning problem, these obstacles are represented as constraints in the objective function. These constraints are defined by Eqs. 5, 6, and 7. These mathematical and visual representations include both inequality and equality constraints to comprehensively characterize the possible obstacles and their implications on the robot’s optimal path.

5 $$\begin{aligned} g_i(x) \ge 0, \quad \text {for } i=1,2,\ldots , m \end{aligned}$$

where ($g_i(x)$) represent the inequality constraints that prevent the robot from entering regions it should avoid, such as obstacles or dangerous zones. They are usually geometric conditions, perhaps involving the robot’s and the obstacles’ shapes and positions.

6 $$\begin{aligned} h_i(x) = 0, \quad \text {for } i=1,2,\ldots , p \end{aligned}$$

The equality constraints ($h_i(x)$) represent conditions that must be satisfied exactly, such as enforcing the robot to pass through specified waypoints along its path.

7 $$\begin{aligned} lb_i \le x_i \le ub_i, \quad \text {for } i=1,2,\ldots , n \end{aligned}$$

Finally, the boundary constraints define the feasible range of each decision variable $x_i$, where $lb_i$ and $ub_i$ denote the lower and upper bounds, respectively. These constraints represent the robot’s physical and operational limitations, such as minimum and maximum allowable speeds or turning angles.

#### Penalty

The penalty term $P(x)$ enforces the constraints. If the robot’s path violates any constraint, an infinite penalty is added to the objective function, effectively excluding that path from consideration.

$$P(x) = {\left\{ \begin{array}{ll} 0 & \text {if } x \in \text {Feasible Region} \\ +\infty & \text {otherwise} \end{array}\right. }$$

In summary, these equations provide a rigorous mathematical framework for solving the robot path planning problem while accounting for multiple objectives and constraints. They are intended to facilitate the algorithm’s search for an optimal or near-optimal path.

#### Path smoothing using cubic spline interpolation

In addressing the operational needs of mobile robotics, it is essential to avoid abrupt turns in the path. This paper introduces an enhancement to the PSOSCANM path planning algorithm by incorporating cubic spline interpolation, as referenced in^31,32^, to achieve a smoother trajectory.

Cubic Spline Interpolation, often shortened to Spline Interpolation, is an efficient method for piecewise interpolation that yields smooth curves. Consider a set of points $\{P = \{p_0, p_1, \ldots , p_n\}, Q = \{q_0, q_1, \ldots , q_n\}\}$, where each $p_i$ corresponds to a $q_i$. The domain of the spline curve is defined within the interval $[p_{\text {min}}, p_{\text {max}}]$, and $n+1$ data points are selected within this range, creating $n$ subintervals. The cubic spline interpolation function, denoted as $Z(p)$, adheres to the following criteria:

1. In each of the $n$ subintervals $[p_i, p_{i+1}]$ (for $i = 0, 1, \ldots , n - 1$), $Z(p)$ is represented by a cubic polynomial. 2. The function satisfies $Z(p_i) = q_i$ for $i = 0, 1, 2, \ldots , n - 1$. 3. Both the first and second derivatives of $Z(p)$ are continuous over the interval $[p_{\text {min}}, p_{\text {max}}]$.

Given the established requirements for cubic spline interpolation, the function $Z(p)$ is formulated from $n$ cubic polynomials as shown in Equation 1:

8 $$\begin{aligned} Z_i(p) = m_i + n_i \cdot (p - p_i) + o_i \cdot (p - p_i)^2 + r_i \cdot (p - p_i)^3 \end{aligned}$$

here, $m_i$, $n_i$, $o_i$, and $r_i$ (for $i = 0, 1, 2, \ldots , n - 1$) are the coefficients to be determined, resulting in $4n$ unknown coefficients in total. The constraint $Z(p_i) = q_i$ for $i = 0, 1, 2, \ldots , n - 1$ is described in Equation 2:

9 $$\begin{aligned} Z(p_{i+1}) = q_{i+1}, \quad m_i = q_i \end{aligned}$$

The step size, defined as $u_i = p_{i+1} - p_i$ for $i = 0, 1, \ldots , n - 2$, leads to the relationship shown in Equation 3:

10 $$\begin{aligned} m_i + n_i \cdot u_i + o_i \cdot u_i^2 + r_i \cdot u_i^3 = q_{i+1} \end{aligned}$$

Continuity of the derivative, a critical aspect of spline interpolation, is expressed in Equation 4:

11 $$\begin{aligned} Z_i'(p_{i+1}) = Z_{i+1}'(p_{i+1}), \quad Z_i''(p_{i+1}) = Z_{i+1}''(p_{i+1}) \quad \text {for } i = 0, 1, 2, \ldots , n - 1 \end{aligned}$$

The function $Z(p)$ is determined by ensuring that it meets the conditions of differential continuity and free boundary constraints. Specifically, the free boundary conditions require that the second derivatives at the interval’s endpoints are zero.

Further, the PSN path is represented as:

$H = \{\text {Start}, (p_1, q_1), (p_2, q_2), \ldots , (p_n, q_n), \text {Goal}\}$, meaning that there are $n + 2$ path nodes, inclusive of the start and goal points. Cubic spline interpolation is applied to these nodes, specifically over the intervals $(p_0, p_1, \ldots , p_{n+1})$ and $(q_0, q_1, \ldots , q_{n+1})$. The resulting path is formed by connecting these adjacent nodes, encompassing the path nodes, interpolation points, as well as the start and goal points.

#### Experiment 1 results and discussion

**Figure 4:**
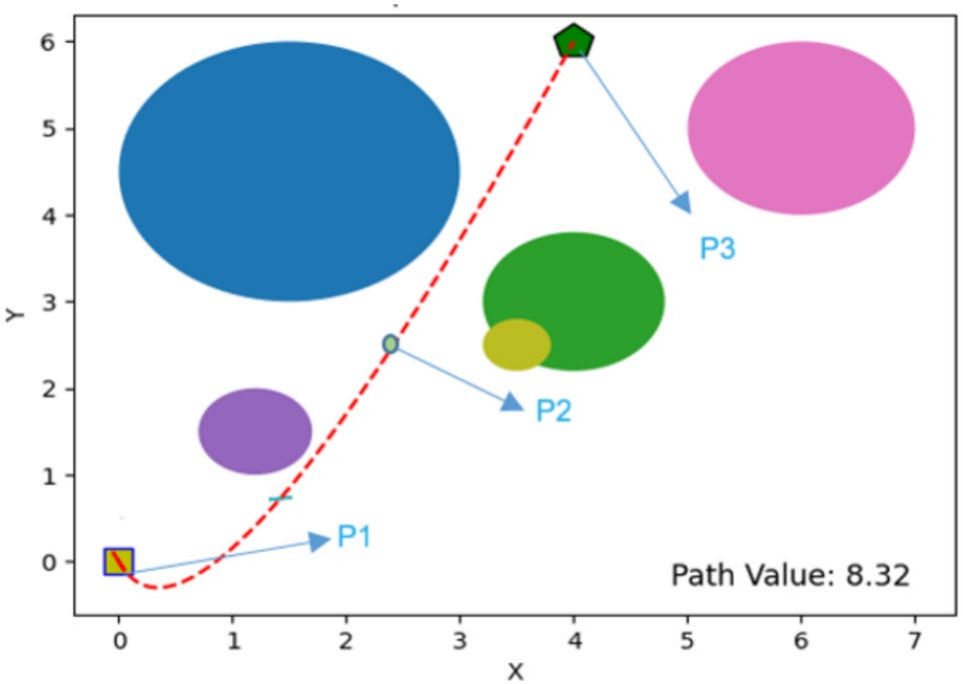
Illustration of optimal path found for environment 1 in experiment 1.

**Figure 5:**
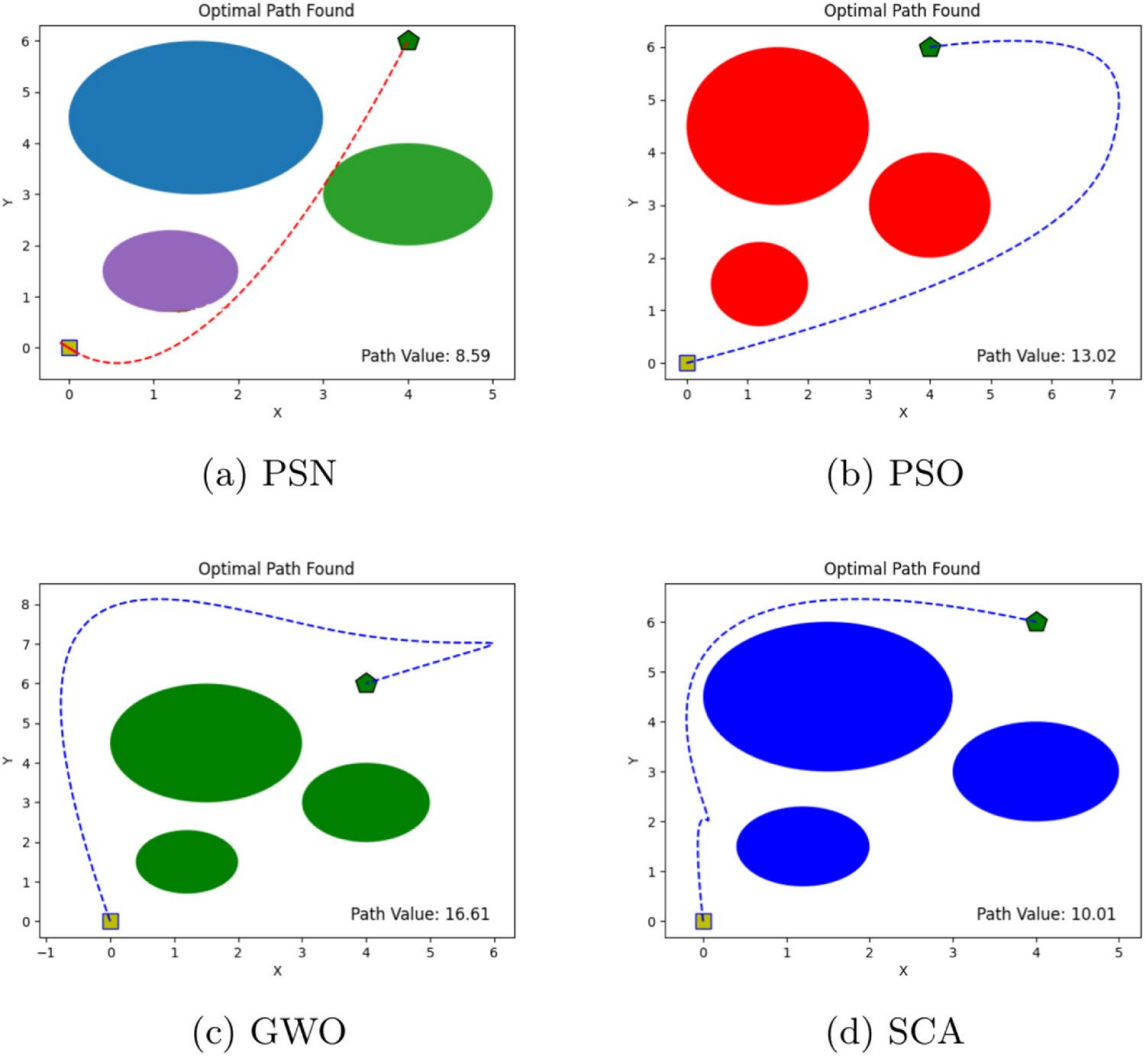
Illustration of optimal path found for environment 2 in experiment 1.

**Figure 6:**
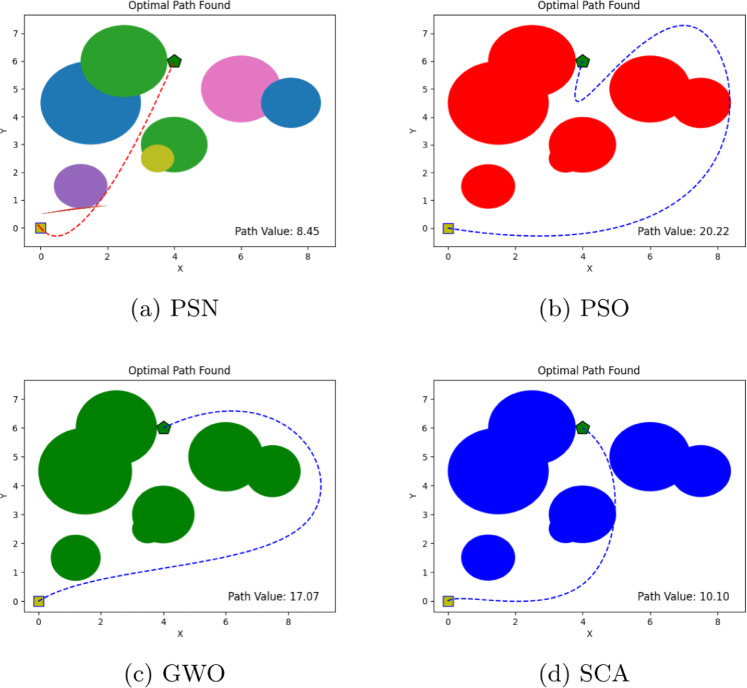
Illustration of optimal path found for environment 3 in experiment 1.

**Figure 7:**
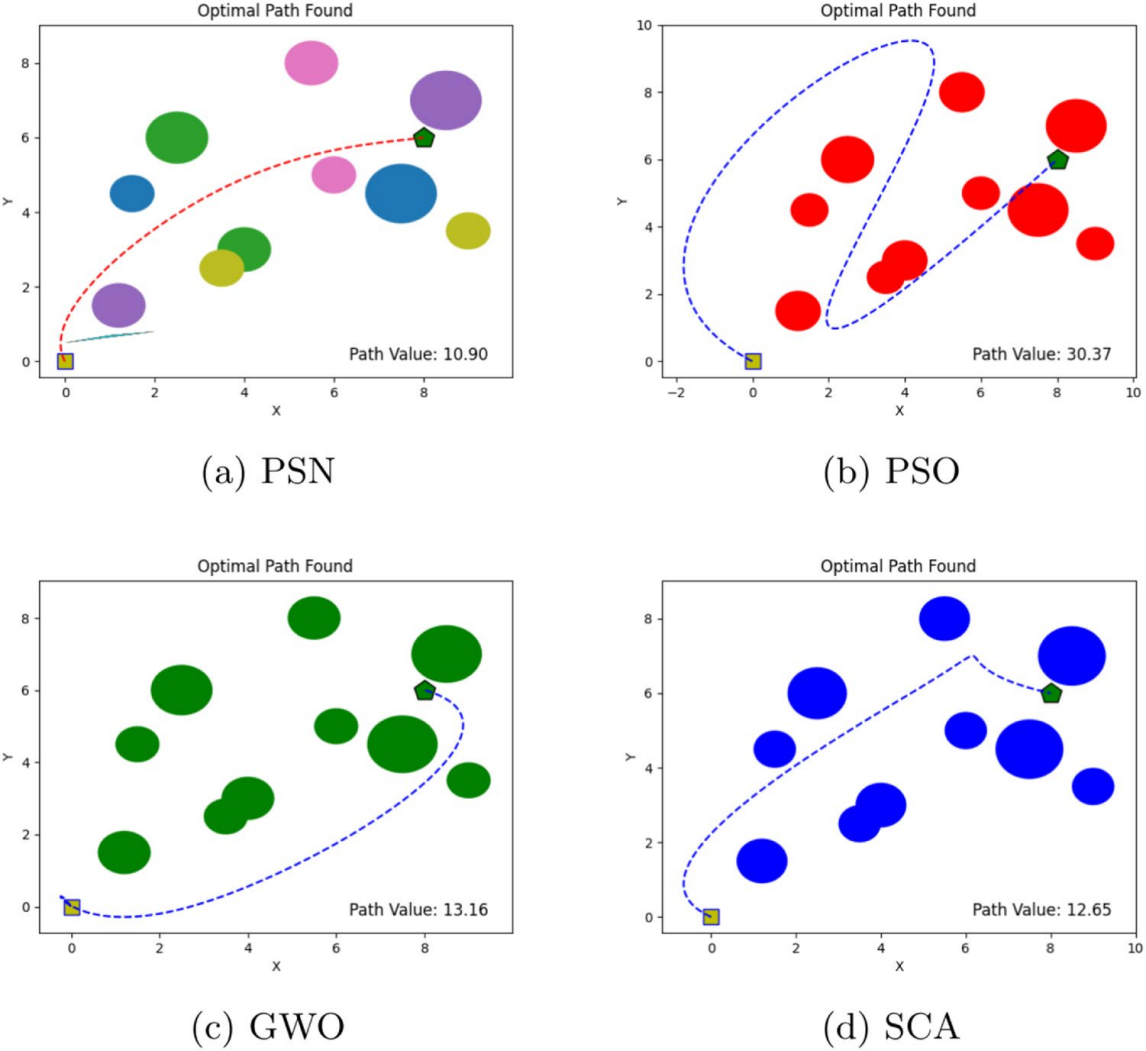
Illustration of optimal path found for environment 4 in experiment 1.

**Figure 8:**
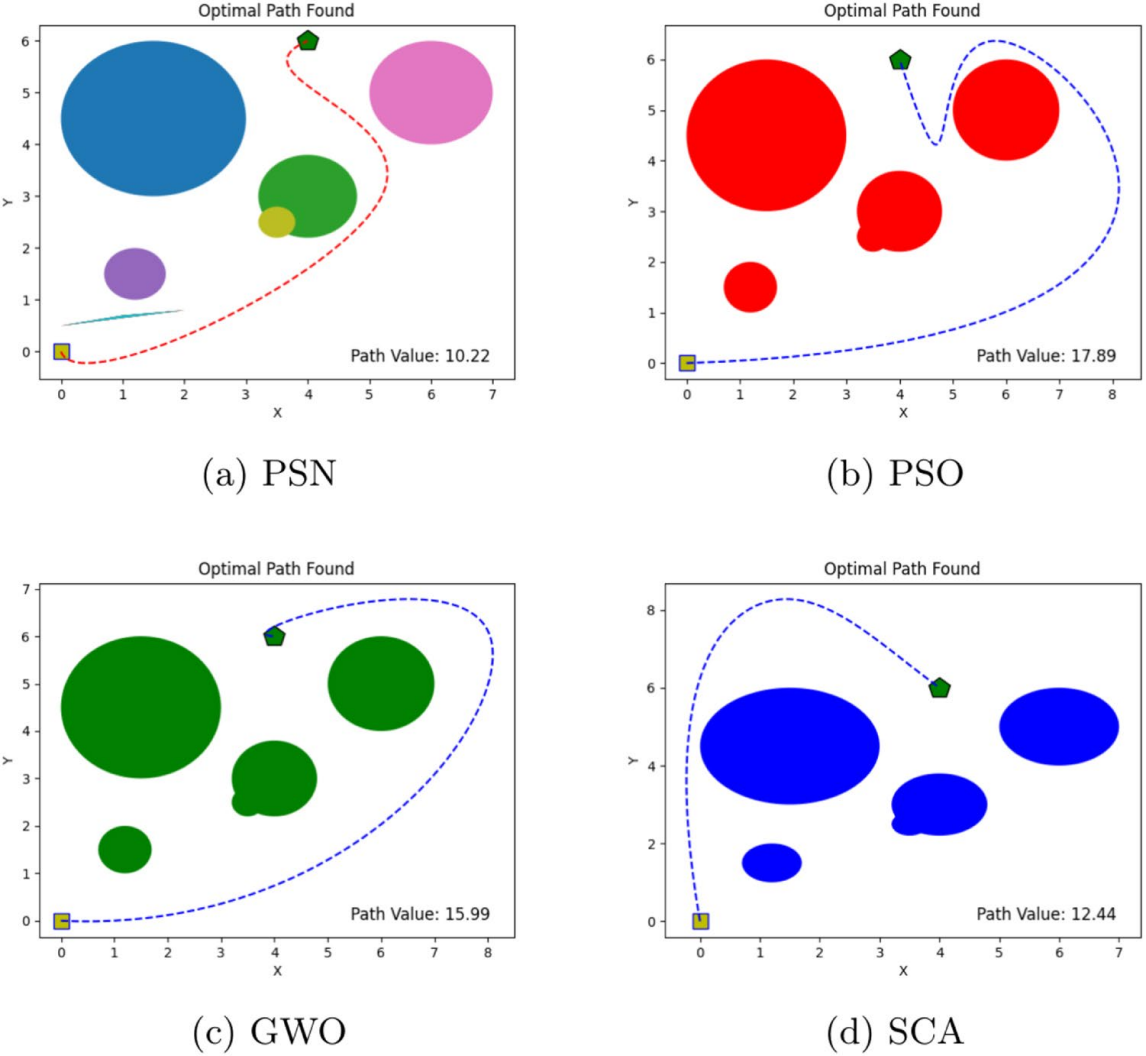
Illustration of optimal path found for environment 5 in experiment 1.

**Figure 9:**
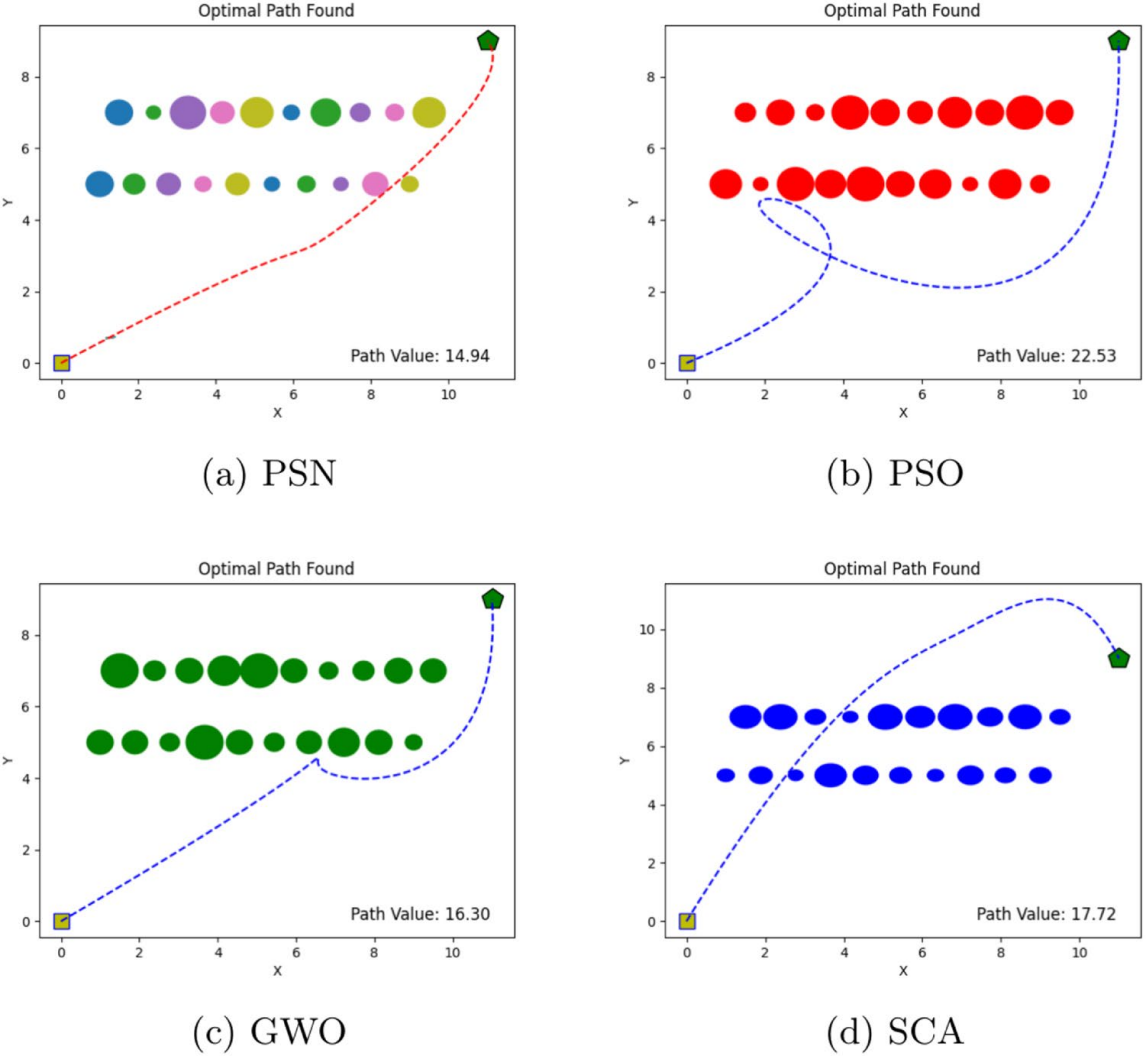
Optimal path found for environment 6 in experiment 1.

The performance of the proposed PSN algorithm was evaluated across multiple scenarios involving both overlapping and discrete obstacles of varying dimensions, as summarized in Table 1. For each environment, comprehensive visualizations are provided, detailing the number of obstacles, the initial and goal positions, and the corresponding optimal path cost values, as illustrated in Figs. 4, 5, 6, 7, 8 and 9. The performance of PSN is benchmarked against several well-established optimisation algorithms, including PSO, SCA, and GWO.

**Table 1:** Comparison of optimal path lengths found by different algorithms.

Exp. No.	# Obstacles	Start point	Target point	PSN	PSO	GWO	SCA
1	3	(0,0)	(4,6)	8.59	13.02	16.61	10.01
2	7	(0,0)	(4,6)	8.45	20.22	17.07	10.10
3	10	(0,0)	(9,6)	10.90	30.37	13.16	12.65
4	5	(0,0)	(4,6)	10.22	17.89	15.99	12.44
5	20	(0,0)	(11,9)	14.94	22.53	16.30	17.72
6	18	(0,0)	(6,6)	9.91	20.28	21.89	18.36

The results shown in Figs. 4, 5, 6, 7, 8 and 9 clearly indicate that PSN consistently outperforms the benchmark algorithms. The objective function employed in these experiments quantifies the overall path cost by jointly considering the travelled distance, obstacle avoidance, and boundary constraints.

Across all evaluated environments, PSN demonstrates robust, reliable performance, regardless of obstacle density or environmental complexity. In **environment 1**, which includes only three obstacles, PSN attained a notably lower path cost of 8.59, compared with 13.02 for PSO, 16.61 for GWO, and 10.01 for SCA. This substantial cost reduction reflects a more efficient trajectory and underscores the effectiveness of the proposed hybrid approach even in relatively simple scenarios.

The efficiency of the PSN algorithm becomes increasingly evident as the number of obstacles grows. In **Environment 2**, which consists of seven obstacles, PSN achieved the lowest path cost of 8.45, significantly outperforming PSO and GWO, which yielded costs of 20.22 and 17.07, respectively. Although SCA achieved the second-best result for 10.10, its performance remained notably inferior to PSN’s. These findings further highlight the strong adaptability and robustness of the proposed hybrid algorithm in handling environments of increased complexity.

Similarly, in **Environment 3**, which contains ten obstacles, PSN obtained a path cost of 10.90, significantly outperforming PSO (30.37) and maintaining a clear advantage over both GWO (13.16) and SCA (12.65). The substantial performance gap between PSN and PSO further demonstrates the hybrid algorithm’s robustness and reduced susceptibility to premature convergence and local optima entrapment, thereby confirming its suitability for complex path-planning scenarios.

Additionally, in **Environment 4** with five distributed obstacles, we observed the same behaviour; the proposed hybrid algorithm outperformed the other methods, achieving the lowest path cost of 10.22, compared to 17.89, 15.99, and 12.44 for PSO, GWO, and SCA, respectively. Despite the lower obstacle density compared to **Environment 3**, PSN’s consistent performance advantage in this moderate-complexity setting highlights its versatility and reliability. **Environment 5** further increased the level of complexity by introducing 20 obstacles arranged in two parallel formations, with the target positioned behind them. In this challenging scenario, PSN identified a path with a cost of 14.94, which is markedly lower than the costs obtained by PSO (22.53), GWO (16.30), and SCA (17.72). This result is particularly noteworthy, as it demonstrates that the proposed algorithm’s performance does not degrade significantly even under substantially increased environmental complexity.

Finally, in **Environment 6**, which includes 18 obstacles, PSN achieved an optimal path cost of 9.91, substantially outperforming PSO (20.28), GWO (21.89), and SCA (18.36). This consistent superiority across all tested environments further validates the effectiveness, robustness, and scalability of the PSN algorithm for robot path-planning tasks in complex static environments.

The outstanding performance of the proposed hybrid PSN algorithm can be justified by its carefully designed structure, which integrates the complementary strengths of PSO, SCA, and the Nelder–Mead Simplex (NMS) method into a unified framework. Each algorithm addresses specific limitations commonly encountered in robot path-planning problems, and their integration yields a robust and efficient optimisation strategy. PSO provides strong global search capabilities by enabling particles to collaboratively explore the solution space. However, despite its effectiveness in identifying promising regions, PSO is prone to premature convergence and may become trapped in local optima, particularly in complex environments with dense obstacles. To mitigate this limitation, SCA is incorporated to enhance both exploration and exploitation processes. Through its sine and cosine update mechanisms, SCA dynamically balances wide-range exploration with focused local refinement, allowing the algorithm to navigate efficiently around obstacles and discover alternative routes when direct paths are obstructed.

Specifically, the sine component of SCA encourages broad exploration of the search space, enabling the robot to investigate multiple potential pathways beyond its immediate surroundings. This capability is especially advantageous in crowded environments where the shortest path may be infeasible due to obstacle interference. Conversely, the cosine component promotes exploitation by refining solutions within promising regions, allowing the robot to optimize its trajectory and identify more efficient or safer paths near an already feasible solution. To enhance local search precision, the NMS method provides effective local optimisation through three core operations: reflection, expansion, and contraction. Reflection allows the algorithm to escape local minima, which frequently occur in obstacle-dense environments where the robot may otherwise remain trapped in suboptimal trajectories. Expansion accelerates convergence by enabling rapid movement toward promising regions once a favourable search direction is identified. Conversely, contraction supports fine-grained solution refinement when navigating narrow passages or approaching the target, thereby ensuring accurate path optimisation and reliable collision avoidance.By combining SCA and NMS within the PSO framework, PSN achieves a robust balance between global exploration and precise local optimization. This synergy enables the algorithm to consistently identify efficient, collision-free paths, even in environments with dense obstacles and complex landscapes, thereby demonstrating its robustness and effectiveness in robot path-planning applications.

#### Sensitivity study: the impact of path node count

The sensitivity study examines the effect of the number of path nodes on the performance of path planning algorithms in a static environment. To support this analysis, a controlled two-dimensional environment was constructed, with both the (x) and (y) coordinates bounded within the interval ([0, 10]). The robot’s starting position is located at ((0,0)), indicated by a red square. In contrast, the target position is set at ((10,10)) and marked by a red “X”. Path nodes are represented by blue circles, and the resulting trajectory is depicted as a red polyline. The experimental setup employs a population size of 15 particles, with the number of path nodes varied from 1 to 10. For each configuration, the algorithm is executed for up to 100 iterations. This setup enables a systematic evaluation of how path discretisation affects solution quality and convergence behaviour. The results obtained for different node configurations are presented in Fig. 11, which illustrates the final paths generated by the PSN algorithm for node counts ranging from 2 to 10. These visual results provide insight into the relationship between the number of path nodes and path-planning performance. Notably, the most efficient paths were achieved when the number of nodes was set to $N=2$ and $N=3$, indicating that an excessively fine discretization does not necessarily lead to improved path quality in this static environment.

### Additional computational evaluation, ablation, and NFE-normalized comparison

Table 2 reports the added ablation results under a fixed iteration budget. On the easier map (Map-S1), PSO+SCA and PSN are effectively tied in mean path length and success rate, while PSO remains slightly worse. On the harder map (Map-S2), the full PSN hybrid attains the best mean path length (13.596) and the highest success rate (66.7%), but also the largest runtime. This directly addresses the reviewers’ concern that path-quality gains should be interpreted jointly with computational cost rather than in isolation.

**Table 2:** Ablation results under a fixed iteration budget (12 seeds, population 30, 100 iterations, 5 internal nodes).

Map	Algorithm	Mean path length	Success rate	Mean runtime (ms)	Mean NFE
Map-S1	PSO	13.224 ± 1.195	75.0%	62.6	3030
Map-S1	PSO+SCA	12.844 ± 0.838	83.3%	121.4	6030
Map-S1	PSN	12.843 ± 0.839	83.3%	353.9	18030
Map-S2	PSO	13.937 ± 0.733	41.7%	88.9	3030
Map-S2	PSO+SCA	13.710 ± 0.563	58.3%	179.7	6030
Map-S2	PSN	13.596 ± 0.363	66.7%	532.8	18030

### Experiment 2: robot path planning in dynamic environment

**Figure 10:**
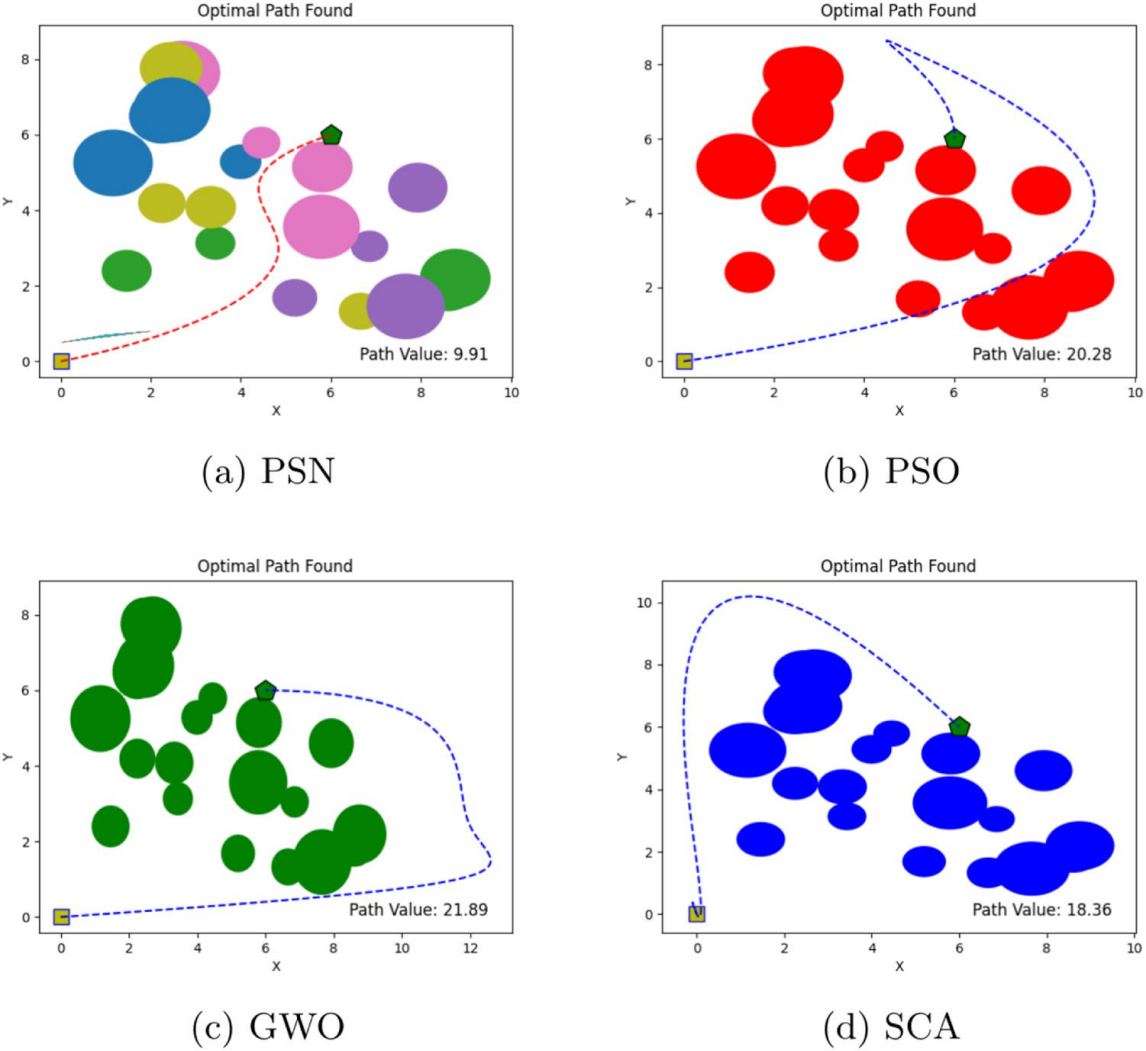
Illustration of optimal path found for experiment 2.

This experiment evaluates the performance of the PSN algorithm in a dynamic environment where both the target and obstacles change positions over time, requiring continuous re-planning. The objective is to generate safe, collision-free trajectories while minimizing travel distance, thereby assessing the robustness and adaptability of PSN under rapidly evolving environmental conditions (Fig. 10).

#### Experimental setup

**Figure 11:**
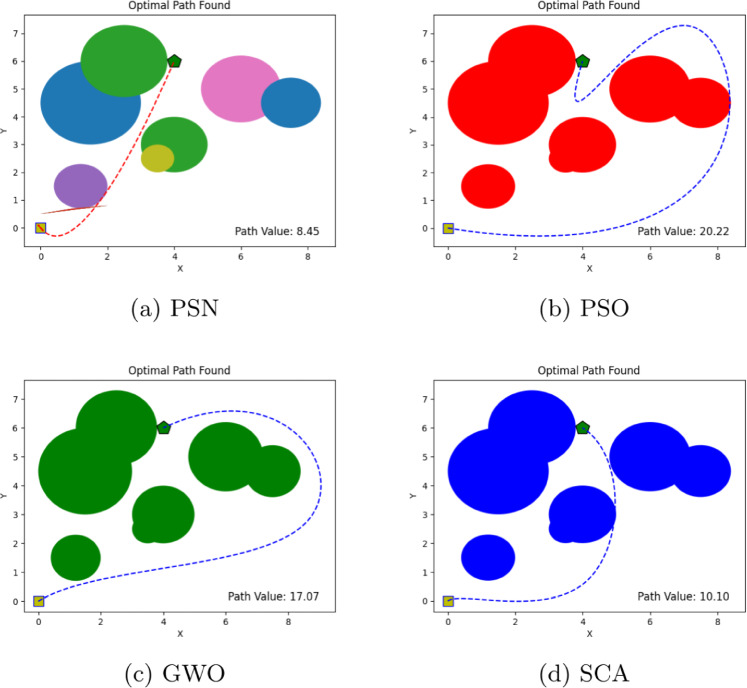
Illustration of optimal path found with N= 2 to N=10.

Path planning in dynamic environments poses substantial challenges due to continuous, often unpredictable variations in obstacle positions and target locations. Unlike static scenarios, autonomous robots operating in such settings must adapt their trajectories in real time, making fast and reliable decisions to ensure both safety and efficiency. Consequently, dynamic path planning requires highly responsive and adaptive strategies capable of managing uncertainty and temporal changes in the environment. Effective navigation in such environments is based on the following assumptions (Fig. 11):

1. The target location and obstacles within the environment are subject to change, requiring the robot to update its path continuously.
2. The robot’s search space and environmental conditions may vary unpredictably, necessitating flexible path planning.
3. Real-time data analysis is critical for the robot to make immediate adjustments to its path.
4. Path planning algorithms must be dynamic, capable of rapid adaptation to new and changing information.
5. Strategies for navigating around moving obstacles must be efficient and proactive, anticipating potential changes in the environment.

To satisfy these requirements, the robot prioritizes the shortest feasible path toward the target while continuously monitoring dynamic obstacles. When potential collisions are detected, the robot employs an adapted version of the hybrid PSN algorithm designed for dynamic environments. This approach enables real-time trajectory recalculation, combining reactive responses with predictive adjustments based on anticipated obstacle movement. As a result, the robot can navigate efficiently and safely through environments that change constantly.

In dynamic autonomous navigation, the fitness function plays a central role in balancing path optimality and feasibility. The fundamental objective remains minimising path length; however, this metric must be continuously updated to reflect changes in obstacle positions and the target location. For a particle ( *P* ), representing a potential path defined by a sequence of cordinates, the path length is computed using Equation 12, as a cumulative Euclidean distance between successive points:

12 $$\begin{aligned} f_{\text {length}}(P) = \sum _{i=0}^{n-1} \sqrt{(x_{i+1} - x_i)^2 + (y_{i+1} - y_i)^2} \end{aligned}$$

In addition to path length, collision risk is evaluated at each path point ($P_i$) with respect to all moving obstacles. This evaluation incorporates the relative positions, radii, and velocities of both the robot and the obstacles, enabling the algorithm to penalize unsafe trajectories.

Within the PSN framework, particle positions and velocities are updated iteratively based on this adaptive fitness function. In dynamic environments, these updates explicitly account for real-time changes in obstacle motion and target location. Consequently, PSN continuously balances efficient path discovery with ongoing environmental adaptation, ensuring that generated paths remain both optimal and feasible throughout execution.

The main constraint is collision avoidance with moving obstacles. Each point ($P_i$) along the candidate path must maintain a safe distance from every obstacle, defined as:

13 $$\begin{aligned} \text {SafeDistance}(P_i, O) = \text {True if } \sqrt{(P_{i,x} - O_x)^2 + (P_{i,y} - O_y)^2} > R_{\text {robot}} + R_{\text {obstacle}} \end{aligned}$$

where (*O*) denotes the obstacle center, and ( $R_{\text {robot}}$ ) and ($R_{\text {obstacle}}$) represent the radius of the robot and obstacle, respectively. A path is considered valid only if this condition holds for all path points and obstacles.

In addition to collision avoidance, the robot must operate within the predefined boundaries of the environment. For an operational region bounded by $(x_{\text {min}}, y_{\text {min}}) ) and ( (x_{\text {max}}, y_{\text {max}})$, this constraint is defined as:

14 $$\begin{aligned} \text {WithinBoundaries}(P_i) = \text {True if } x_{\text {min}} \le P_{i,x} \le x_{\text {max}} \text { and } y_{\text {min}} \le P_{i,y} \le y_{\text {max}} \end{aligned}$$

This constraint ensures that each point $P_i$ of the path lies within the specified boundaries of the environment.

Furthermore we conducted expirements that include a quantitative replanning benchmark with 10 random episodes and reports success rate, collision rate, mean latency, 95th-percentile latency, travelled distance, and mean number of control steps (Table 3).

**Table 3:** Quantitative dynamic replanning results over 10 random episodes.

Algorithm	Success	Collision	Mean latency (ms)	P95 latency (ms)	Mean travelled distance	Mean steps
PSO	10.0%	90.0%	14.8	15.7	5.37	21.5
PSO+SCA	20.0%	80.0%	30.2	31.6	5.47	21.9
PSN	40.0%	60.0%	89.0	98.0	6.62	26.5

**Figure 12:**
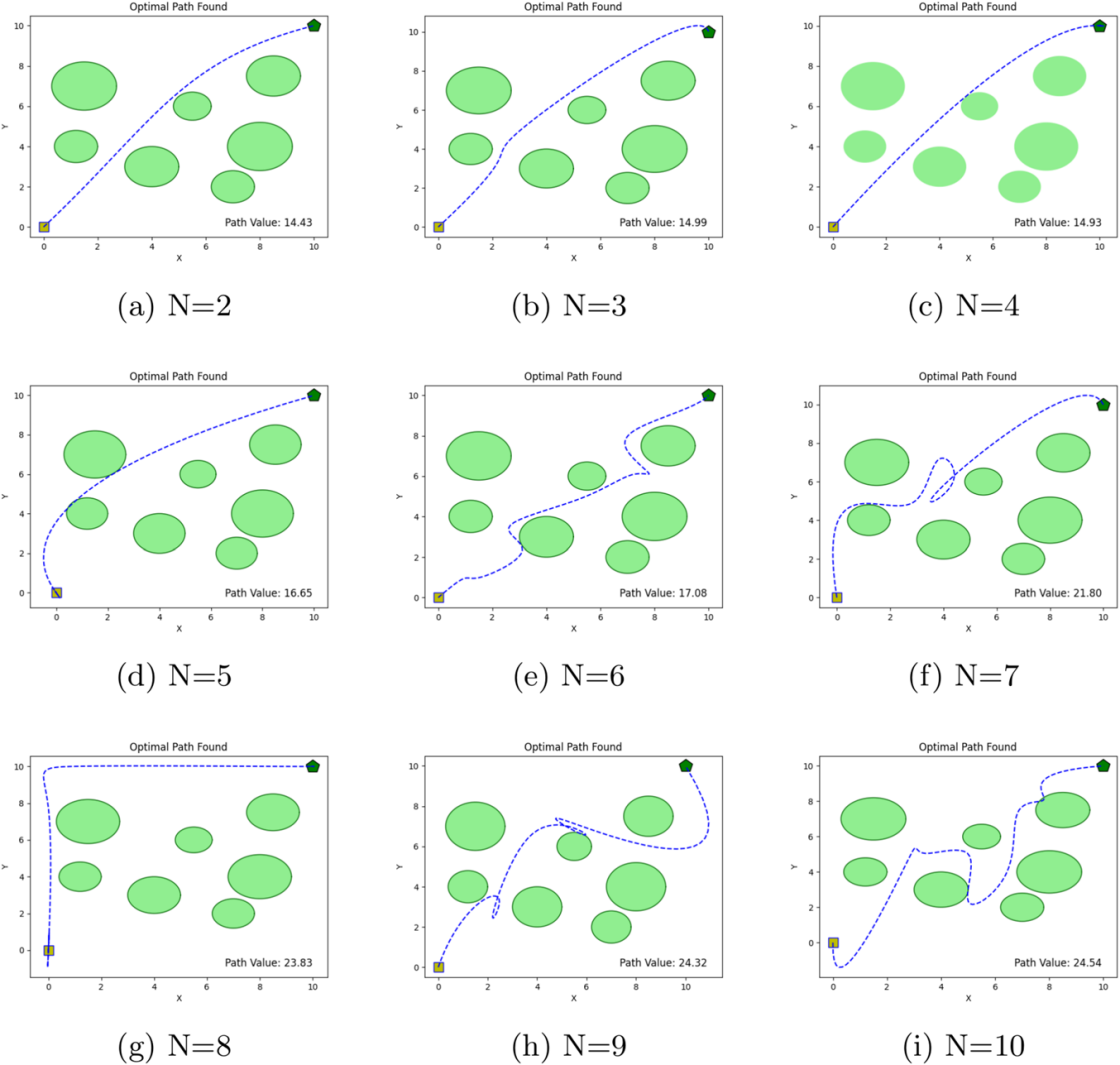
Representative successful PSN dynamic episode from the revision-only benchmark (seed 0).

The dynamic results reveal the same trade-off observed in the static study: PSN delivers the highest success rate (40%) and the lowest collision rate (60%), but at a substantially higher mean replanning latency (88.96 ms) than PSO+SCA (30.16 ms) and PSO (14.79 ms). This makes the real-time implication explicit instead of implicit (Fig. 12).

**Figure 13:**
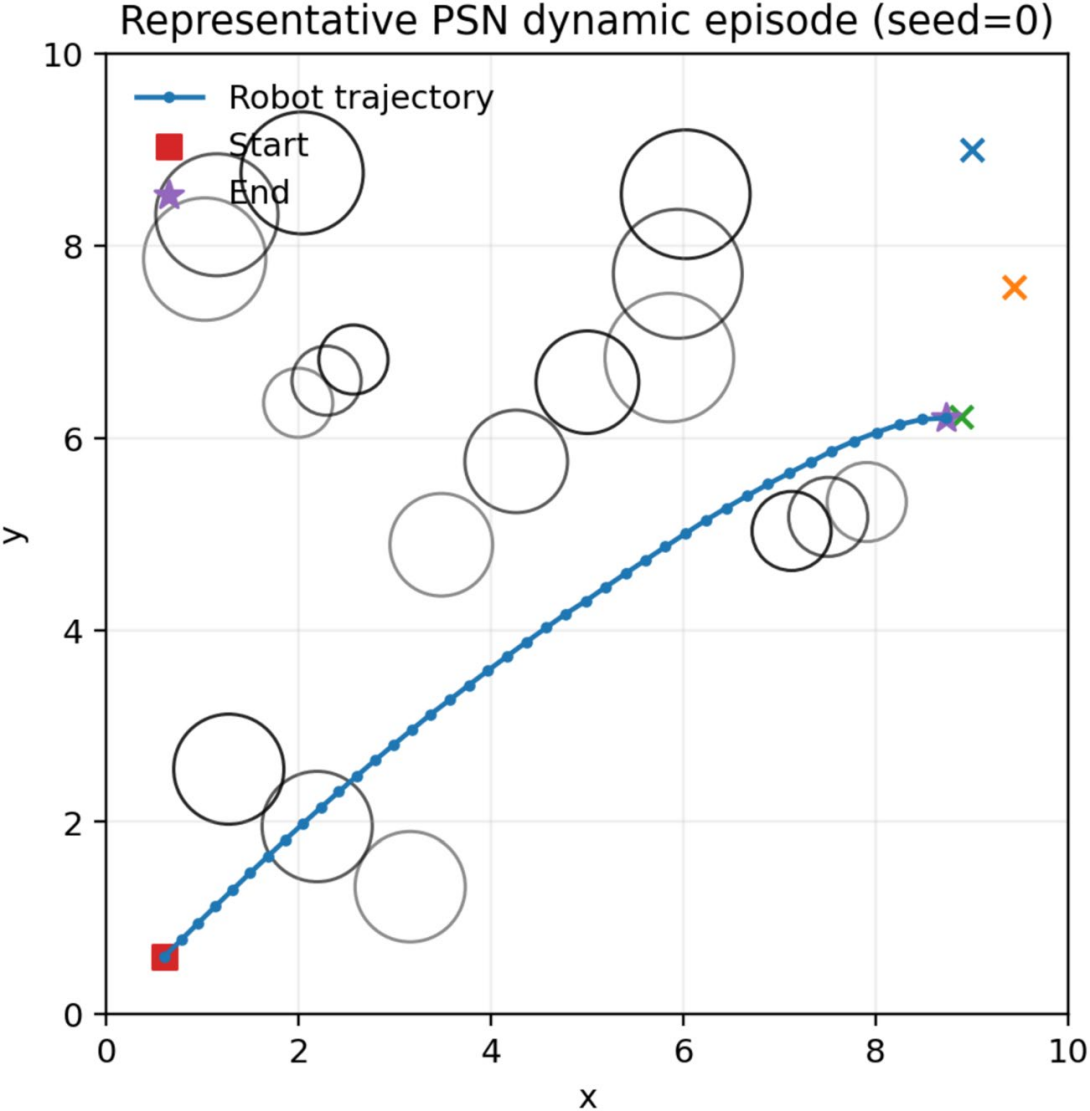
NFE-normalized convergence comparison on Map-S2 (mean ± standard deviation across 12 seeds).

Because PSN evaluates more candidates per iteration than the simpler baselines, Table 4 repeats the comparison under an approximately equal NFE budget of 9000 evaluations. Under this normalization, PSN remains tied-best on Map-S1 and attains the best mean path length on Map-S2 (13.589 versus 13.607 for PSO+SCA and 13.937 for PSO), but PSO+SCA achieves the highest success rate on the harder map. This result shows that the full hybrid improves average path quality, yet the extra local-search effort does not dominate every metric once evaluation counts are equalized (Fig. 13).

**Table 4:** Ablation results under an equal-NFE budget of approximately 9000 function evaluations.

Map	Algorithm	Mean path length	Success rate	Mean runtime (ms)	Mean NFE
Map-S1	PSO	13.224 ± 1.195	75.0%	181.6	9000
Map-S1	PSO+SCA	12.843 ± 0.839	83.3%	190.5	8970
Map-S1	PSN	12.844 ± 0.839	83.3%	177.2	8850
Map-S2	PSO	13.937 ± 0.733	41.7%	268.0	9000
Map-S2	PSO+SCA	13.607 ± 0.491	58.3%	264.7	8970
Map-S2	PSN	13.589 ± 0.361	41.7%	271.4	8850

**Figure 14:**
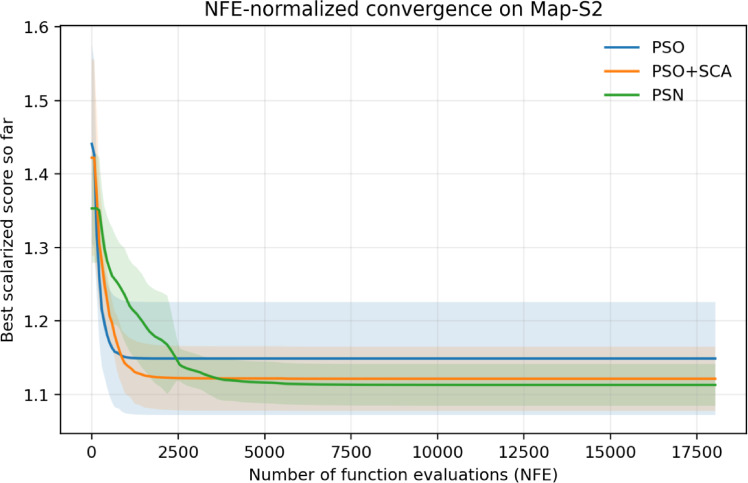
Representative benchmark maps used for the ablation study. The plotted trajectories are the best PSN runs on Map-S1 and Map-S2.

The stage-wise runtime breakdown on Map-S2 shows that, on average, the PSO stage accounts for 16.3% of execution time (86.3 ms), the trigonometric exploration stage accounts for 34.1% (179.9 ms), and the simplex-inspired local refinement stage accounts for 49.6% (261.9 ms). This breakdown explains why the PSN gains are accompanied by noticeably larger latency and further justifies the NFE-normalized evaluation (Fig. 14).

#### Experiment 2 results and discussion

In this experiment, the robot is initialised at the origin (0,0) and aims to reach a moving target whose initial position is set to (0,10). Unlike static scenarios, the target’s location changes randomly over time, introducing additional complexity and requiring continuous trajectory adaptation. The environment contains eight dynamic obstacles with randomly assigned properties, including position, size, and velocity. Each obstacle’s radius is randomly selected within the range of 0.3 to 1.5 units, while its initial position is uniformly distributed within the interior of the operational domain to avoid boundary interference.

The motion of each obstacle is governed by a constant velocity vector, with both horizontal and vertical components randomly chosen within the interval ([-0.05, 0.05]) units per time step. These settings result in diverse and unpredictable obstacle trajectories throughout the simulation. Path planning is performed using the PSN algorithm, which enables the robot to navigate toward the moving target while continuously accounting for obstacle motion. At each simulation step, the robot recalculates its trajectory based on the current positions of the target and obstacles. When a potential collision is detected, the algorithm dynamically adjusts the robot’s direction and step size to generate an alternative collision-free path, ensuring safe navigation in real time.

**Figure 15:**
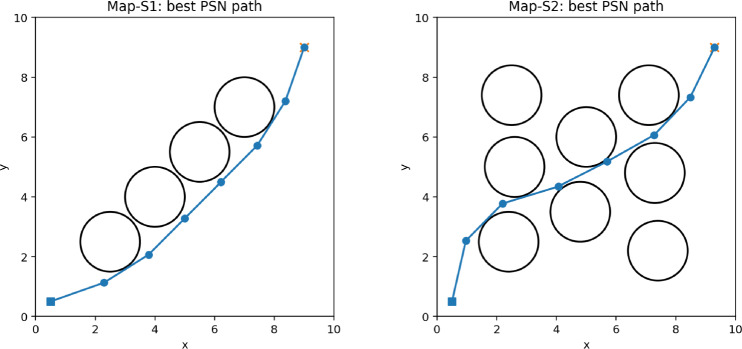
Illustration of optimal path found for experiment 5.

The dynamic environment, characterised by the simultaneous movement of both obstacles and the target, requires constant adaptation of the robot’s path. This continuous re-planning process highlights the algorithm’s ability to respond effectively to rapidly changing environmental conditions. Throughout the simulation, the robot’s total distance travelled is incrementally computed and updated. The robot’s trajectory is visualised as a sequence of connected line segments, providing a real-time depiction of its motion and adaptation. Overall, this experiment illustrates the inherent challenges of real-time path planning in dynamic environments, where neither obstacles nor targets remain stationary. The stochastic movement patterns of both entities create a highly demanding scenario that rigorously tests the PSN algorithm’s robustness, adaptability, and effectiveness in collision avoidance and trajectory optimization (Fig. 15).

## Conclusion and future works

This paper presented SHARP, a hybrid metaheuristic approach for intelligent robotic path planning. The proposed framework builds on a PSN search mechanism that combines the global exploration capability of Particle Swarm Optimization, the directional exploration and exploitation behavior of the Sine Cosine Algorithm, and a lightweight simplex-inspired refinement stage based on Nelder–Mead operations. On top of this shared search mechanism, two scalarization-based multi-criteria planning variants were introduced: Priority-PSN and No-Preference-PSN. Priority-PSN emphasizes path-length minimization while penalizing obstacle and boundary violations, whereas No-Preference-PSN selects a compromise solution by minimizing the normalized distance to an ideal point. In addition, cubic-spline interpolation was used to improve the smoothness and executability of the final waypoint-based trajectory.

The experimental results demonstrate that SHARP is effective for static robotic path planning in cluttered two-dimensional environments. Across the six static benchmark environments, the proposed PSN-based planner consistently achieved lower path costs than PSO, GWO, and SCA, showing its ability to identify shorter collision-free trajectories under different obstacle densities. The sensitivity study further indicated that increasing the number of intermediate path nodes does not always improve solution quality, suggesting that path representation should be selected carefully to balance flexibility, smoothness, and search complexity.

The dynamic replanning experiment further confirms the adaptability of the proposed approach in environments with moving obstacles and a moving target. PSN achieved the highest success rate among the evaluated variants and reduced the collision rate compared with PSO and PSO+SCA. However, this improvement was obtained at the cost of higher replanning latency. This result highlights an important practical trade-off: the additional search and refinement stages improve robustness, but they also increase computational overhead. Consequently, real-time deployment requires careful parameter selection and possibly further acceleration.

Future work will focus on improving the computational efficiency of SHARP for real-time robotic navigation. Promising directions include adaptive control of the number of PSN refinement steps, early-stopping strategies, parallel implementation, and dynamic adjustment of penalty parameters according to obstacle density and collision risk. Future studies should also evaluate the proposed approach in larger and more realistic environments, including non-circular obstacles, uncertain sensor measurements, nonholonomic robot constraints, and three-dimensional workspaces. Finally, implementing SHARP on physical robotic platforms would provide stronger evidence of its practical applicability and allow evaluation under real sensing, actuation, and timing constraints.

## Data Availability

The datasets generated and/or analysed during the current study are available upon request.

## Declarations

### The use of AI

The authors declare that ChatGPT was used to improve the language, grammar, and clarity of the manuscript. The authors take full responsibility for the content of this work.
